# Variations in External and Internal Intensities and Impact of Maturational Age on Soccer Training Tasks

**DOI:** 10.3390/s24175656

**Published:** 2024-08-30

**Authors:** Juan Manuel García-Ceberino, José Manuel Cantonero-Cobos, Cristina Conde, Eduardo José Fernández-Ozcorta

**Affiliations:** 1 Facultad de Educación, Psicología y Ciencias del Deporte, Universidad de Huelva, Av. de las Fuerzas Armadas s/n, 21007 Huelva, Spain; josemanuel.cantonero@ddi.uhu.es (J.M.C.-C.); cristina.conde@dempc.uhu.es (C.C.); eduardo.fernandez@dempc.uhu.es (E.J.F.-O.); 2 Grupo de Optimización del Entrenamiento y Rendimiento Deportivo (GOERD), Facultad de Ciencias del Deporte, Universidad de Extremadura, Av. de la Universidad s/n, 10001 Cáceres, Spain; 3 Grupo EMOTION, Facultad de Educación, Psicología y Ciencias del Deporte, Universidad de Huelva, Av. de las Fuerzas Armadas s/n, 21007 Huelva, Spain

**Keywords:** physical-physiological demand, inertial movement unit, peak height velocity, maturity offset

## Abstract

During peak height velocity, adjusting training intensity is crucial for optimizing performance and minimizing injury risk. This cross-sectional study compares external and internal intensities in different training tasks (analytical tasks, small-sided games, and training matches) and analyzes their effect on the maturation age of young players. Fifty-five U-15 and U-16 boys from two soccer clubs in southwestern Spain were monitored using inertial movement units and heart rate monitors to report training intensities. Anthropometric data and birthdates were collected to estimate maturation age. The Friedman test and Durbin–Conover post hoc test identified specific differences between groups, and Spearman’s rank correlation coefficients assessed variable impacts. Training matches showed significantly higher distance covered, maximum and average speed, and average heart rate compared to small-sided games and analytical tasks. High-intensity actions and sprints were significantly higher (*p* < 0.0001) during training matches compared to analytical tasks and during small-sided games compared to analytical tasks. Player load per minute was significantly highest (*p* < 0.05) during training matches, followed by small-sided games, and lowest in analytical tasks. Positive correlations between maturational age and high-intensity actions, accelerations, and decelerations indicated higher intensity (*p* < 0.05) in more mature players. A negative correlation between player load per minute and maturational age suggested more efficient intensity management in mature players. These findings highlight the importance of considering biological maturation and training task variability in youth athletes’ development.

## 1. Introduction

Training young athletes, especially in demanding disciplines such as soccer, requires a meticulous and adaptive approach that considers variations in biological maturity. This period of accelerated growth, known as the peak height velocity (PHV) [1], is critical as athletes experience rapid physical–physiological development that can significantly impact their performance and injury risk. It is essential to study intensity variables in different training tasks with respect to PHV to optimize performance and minimize risks.

### 1.1. Training Intensities in Sports

Staunton et al. [2] replaced the term “load” with “intensity” in sport and exercise science; therefore, this term is used. Proper training planning involves understanding the distribution of the intensities caused by different training tasks to effectively manage their impact on players’ development and performance [3]. Training intensities are classified into external (eTL) and internal (iTL) intensity [4]. The eTL represents the physical demands endured by players during training or competition [5]. The eTL can be measured objectively by inertial movement units (IMUs), which record kinematic (distances covered in meters, accelerations, decelerations, different ranges of speeds, etc.) and neuromuscular (impacts, player load, etc.) variables [6,7], and subjectively by the Integral Analysis System of Training Tasks (SIATE) in invasion games [8]. On the other hand, the iTL refers to the physiological response elicited by the eTL, and it can be assessed objectively through physiological instruments such as heart rate (HR) monitoring, lactate levels, or maximal oxygen consumption [9]. Subjective iTL (psycho-physiological demand) can be calculated using the Rating of Perception Effort scale proposed by Borg [10].

It is important to take an integrated approach to training intensities, where eTL and iTL are used in combination to better understand training stress. For example, athletes repeating exactly the same session on different days may maintain the same eTL but experience quite different iTL (HR, blood lactate, perception of effort, etc.) depending on their fatigue state, emotional disorders, etc. [4]. Today, devices that include systems with IMUs and HR monitors objectively measure these intensities (eTL and iTL). They integrate sensors such as triaxial accelerometers, gyroscopes, magnetometers, etc. They also incorporate global positioning systems and ultra-wideband technology [11]. This technology has been used in team sports [12,13,14,15] and, specifically, in elite [16,17,18], semi-professional [19,20], amateur [21], and training soccer [22,23,24,25,26]. The study of intensities in soccer is common since it is considered a team sport with high-intensity intermittent efforts [27].

### 1.2. Soccer Training Tasks

Depending on the objectives of each training session, coaches manipulate the constraints of the training tasks [8], resulting in different types of training tasks: analytical tasks, small-sided games (SSGs), and full games. Bangsbo et al. [28] define these three types of training tasks. Analytical tasks focus on the development of specific technical skills (e.g., passing, ball control, shooting, or defensive tactics) in an isolated and controlled context, i.e., outside of a full match environment. SSGs are played with a reduced number of players, on a smaller than usual playing field and/or with modified rules. They simulate game situations with fewer players, allowing for increased contact with the ball and more opportunities to make decisions under pressure during the game (e.g., situations 2 vs. 1, 3 vs. 2, 4 vs. 2, etc., in reduced fields). Full games are matches played with a standard number of players (7 vs. 7, 8 vs. 8, or 11 vs. 11, depending on the category) on a regulation-size soccer field. These matches represent the actual competitive situation and are essential for the tactical development and physical endurance of the players. SSGs have become one of the most used tasks by coaches in soccer training indifferent categories and levels. Thus, research on their effect on training sessions is becoming increasingly frequent [22,29,30].

This manipulation of training task constraints results in different intensities for young players. In this regard, Gómez-Carmona et al. [22] monitored U-18 soccer players, using IMUs, during official matches and SSGs with different objectives (SSG1: keep the ball; SSG2: keep the ball and progress; SSG3: keep the ball, progress, and finish in mini-goals; and SSG4: keep the ball, progress, and finish in an official goal with goalkeeper). Only SSG4 presents similar demands to those of the competition. Machado et al. [24] report that manipulation of training tasks constraints affects the physical and tactical demands in two 4 vs. 4 SSGs for U-17 recreational soccer players: Structural SSG, in which the field format and goal size were manipulated, and Functional SSG, in which players were allowed to kick the ball twice and at least five passes to shoot at the opponent’s goal. Players covered more distance in sprinting and high-speed running, in addition to better exploring the game space in the Structural SSG. In the Functional SSG, players were closer to the ball and their teammates, decreasing the effective game space [24]. Clemente et al. [25] compared the influence of age-level groups (i.e., U-13, U-15, and U-18) during 4 vs. 4 plus goalkeepers SSGs, finding that older players occupied larger areas, greater mean distance between teams centroids, and greater distance between players and the stretch index.

### 1.3. Maturation Age in Young Players

PHV marks a turning point in the physical–physiological development of players, where significant changes in height, body mass, and muscle composition occur. During this period, these changes can influence both sports performance and susceptibility to injuries. Research has shown that players in the PHV phase are more prone to injuries due to the combination of rapid growth and increased training intensity. For instance, players in the PHV period have a significantly higher injury load, especially those related to growth [31]. To properly manage training intensities during PHV, it is crucial to adjust the eTL, which encompasses factors such as intensity, volume, and training frequency. This careful management is essential to preventing overtraining and reducing the risk of injuries. Towlson et al. [32] emphasize the importance of adapting training intensities according to individual biological maturity to optimize performance and minimize risks. Additionally, motor skills such as explosive strength and running speed reach their maximum development during PHV, suggesting that training intensities should be adjusted to maximize performance during this period while simultaneously avoiding excessive fatigue and injuries [33]. Bult et al. [34] also reinforce the importance of adjusting training intensities during this critical period by highlighting the higher risk and load of injuries in players in the PHV period. Furthermore, Dupré and Potthast [35] investigated how PHV influences hip joint kinematics and adductor muscle forces in adolescent soccer players. Their results indicate that changes in movement technique during PHV can increase the risk of groin injuries, highlighting the need to monitor and adjust training intensities during this period.

### 1.4. Objective and Hypothesis of This Study

The relevance and necessity of an adaptive approach in training young players, particularly regarding PHV, are clear. This critical period of physical–physiological development significantly impacts players’ performance and health. The cited scientific evidence shows that training intensities must be carefully adjusted to avoid injuries and maximize physical–physiological development. Furthermore, the importance of individualized training that considers each player’s biological maturity is emphasized. This perspective not only enhances sports performance but also protects the physical–physiological integrity of young players during their growth. This study aimed to monitor and compare eTL and iTL according to the type of training task (i.e., analytical tasks, SSGs, and training matches). Similarly, the effect of these training tasks on the maturation age of young soccer players was analyzed. We hypothesized the following: (1) higher cognitive involvement in tasks will lead to higher eTL and iTL values in players; and (ii) maturational age will influence both eTL and iTL during training tasks, with players in the PHV period experiencing higher eTL and iTL values compared to players who have either passed or have not yet reached this critical developmental phase.

## 2. Materials and Methods

### 2.1. Study Design

An empirical study was conducted using a comparative and cross-sectional associative strategy [36] in a natural sport context without manipulation of the variables by the researchers. Ethical guidelines were followed according to the Declaration of Helsinki of 1975 (with subsequent amendments) and Organic Law 3/2018, of December 5, on the protection of personal data in research and the guarantee of digital rights, to ensure the ethical considerations of scientific research involving human subjects. This study received approval from the University Bioethics Committee for Biomedical Research (SICEIA-2024-001206).

### 2.2. Population, Participants, and Sample

Formative soccer players were analyzed. Fifty-five U-15 and U-16 boys (age: 15.54 ± 0.29 years; weight: 62.83 ± 8.06 kg; height: 1.72 ± 0.06 m; sitting height: 87.55 ± 3.70 cm; body mass index: 21.28 ± 2.07 kg/m), representing two soccer clubs from southwestern Spain, participated in this study. Each club was represented by two teams in this category (A and B). Players engaged in an average of 6–8 h of soccer training and competitive play per week. Injured players were excluded from this study. Goalkeepers were also excluded because their physical–physiological intensity differs from all other field players.

The G*Power v.3.1 software indicated a required sample size of 55 subjects with an effect size = 0.20, α error = 0.05, and power = 0.90. A convenience sampling method was used, selecting those clubs that authorized this study; in this case, two clubs participated, and both were located within 30 km of the lead researcher. Studies focused on coaches’ actions on fields and courts in team sports often employ small sample sizes. However, this does not imply that the scientific knowledge generated is invalid or incapable of effectively communicating the results to the scientific community. In this regard, our study meets the criteria established by Lago et al. [37] for generating scientific knowledge: (1) complementing statistical significance with magnitude-based inferences (e.g., effect size); (2) increasing the number of observations whenever possible; and (3) seeking to create scientific laws to explain players’ behavior (Merton’s middle-range theory).

### 2.3. Variables and Instruments

Anthropometry. A portable stadiometer (SECA 213, Hamburg, Germany) was used to measure height, and a 40 cm plyometric box was used to measure sitting height. Lastly, weight was obtained using a portable digital scale (SECA 813, Hamburg, Germany).

Intensity. Each player was equipped with an IMU with global positioning systems (WIMU PRO^TM^; RealTrack Systems S.L., Almería, Spain; now part of Hudl, Lincoln, NE, USA) and a GARMIN^TM^ HR pectoral band (Garmin Ltd., Olathe, KS, USA). Both devices were synchronized with each other using ANT+ methodology. This monitoring system has demonstrated high reliability and validity for data collection in outdoor spaces [38]. Data were retrieved by S-PRO^TM^ v.990 software (RealTrack Systems S.L., Almería, Spain; now part of Hudl, USA) for further analysis in statistical software. The eTL (kinematic and neuromuscular) and iTL (HR) intensities analyzed are shown in Table 1.

### 2.4. Procedure

Firstly, the different clubs were contacted with the purpose of informing them about this study’s objectives, thus inviting them to participate in it. Once permission was obtained, the coaches and players of the respective teams were informed.

The next step was to take the anthropometric measurements of the players. Using the guidelines from the International Society for the Advancement of Kinanthropometry [39], the following measurements were taken: standing height, sitting height using a stadiometer, and body mass using a portable digital scale. Sports science professionals familiar with gathering such data in similar settings collected anthropometric data. First, both the stadiometer and the scale were calibrated correctly on a completely horizontal surface. In the second phase, participants were asked to remove their shoes and step onto the scale to determine their body mass (kg). Third, participants were instructed to stand on the stadiometer, also barefoot, with their head uncovered and feet together. It was ensured that their heels, buttocks, shoulders, and the back of the head (with the head in the Frankfurt plane, meaning the auditory meatus and the lower edge of the eye orbit should be in a horizontal plane) were in contact with the stadiometer to determine standing height (cm). Finally, the portable stadiometer was placed on a 40 cm high base, and participants were instructed to sit on the elevated stadiometer base with their back against the back of the stadiometer, applying the same upper body positioning strategy as described above. The leg length was calculated by subtracting the sitting height from the standing height. After obtaining all these data, biological maturity status was estimated using the Fransen et al. [40] method. In addition to the anthropometric data (i.e., standing height, sitting height, and body mass), the equation required the date of birth and the date of measurement. These data were entered into a specially designed Excel sheet that, using a multiple regression equation, provided an estimate of the proximity to PHV expressed in years from PHV. A score of “0” indicates that an individual is at PHV. To further improve the precision of estimates for age at PHV, the method proposed by Fransen et al. [40] was utilized. This method uses a maturity ratio (chronological age/age at PHV) rather than maturity offset (chronological age/age at PHV), as it is considered a more appropriate representation of the non-linear relationship between anthropometric variables and maturity offset. This variable is referred to as Offset.

Then, a training session of between 60 and 120 min, for each team, was monitored during the competitive period of the 2023/2024 season. The coaches decided on the day the researchers would attend the training field (outdoor). The monitoring procedure was as follows: (1) arrival of the research team at the training field 45 min before for the activation and synchronization of the IMUs (WIMU PRO^TM^). Due to the simultaneous use of different IMUs, a synchronization process, according to the recommendations established by Gómez-Carmona et al. [41], was necessary to keep the data on the same timeline; (2) summon the players 30 min before to equip them with an anatomical harness at the scapular level, in which the inertial device was incorporated, along with a thoracic HR monitor; (3) once the previous steps were completed, the training session began as normal. The coaches had freedom in the design and organization of the tasks, but the session had to have the following structure: warm-up → analytical part → SSGs (keep the ball, progress, and finish in an official goal with goalkeeper) → training match → cool down. Therefore, the players were familiar with the training tasks. During each training session, the researchers recorded the intervals of each task, using the specialized S-VIVO^TM^ v.807 software (RealTrack Systems S.L., Almería, Spain; now part of Hudl, USA) that allows the data to be viewed in real time, to exclude pauses (e.g., hydration, feedback, etc.) where there was no motor participation. Once each training session was completed, (4) the data were downloaded to a laptop computer for further analysis using S-PRO^TM^ software. An informative dossier of each training session was prepared and delivered to the coaches.

**Figure 1:**
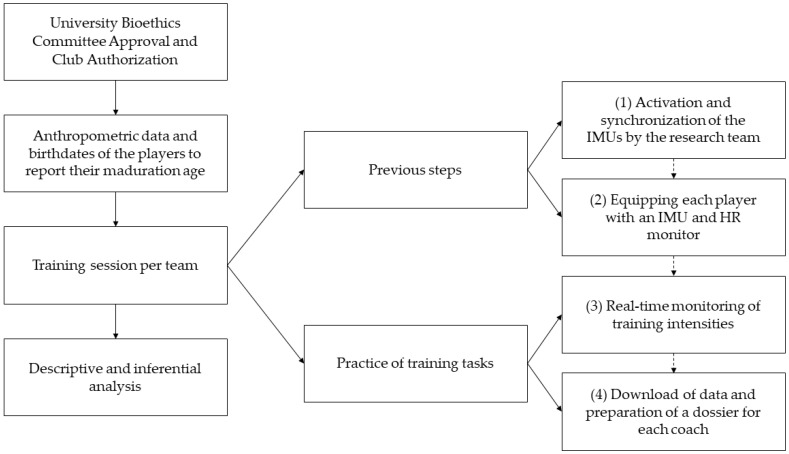
Study design and procedure. Note: IMUs = inertial movement units; HR = heart rate.

Finally, the descriptive and inferential analyses of the PHV data and training intensities were performed. Figure 1 summarizes the study design and procedure.

### 2.5. Statistical Analysis

To analyze the data, a series of statistical tests was performed to compare the different conditions across various physical–physiological variables. The analysis was carried out in several steps, each designed to ensure the robustness and clarity of the results. First, descriptive statistics were calculated for all variables to summarize the data. These statistics included measures of central tendency (mean and median) and variability (standard deviation and interquartile range). This step provided an overview of the data distribution and highlighted any potential outliers or trends. Next, the data were tested for normality using the Shapiro–Wilk test. This test indicated that the data did not follow a normal distribution (*p* < 0.05), requiring the use of non-parametric tests for subsequent analyses.

Due to the non-parametric nature of the data, the Friedman test was chosen to evaluate the overall differences between the conditions. The Friedman test is a non-parametric alternative to the repeated measures ANOVA and is used to detect differences in treatments across multiple test attempts. Upon obtaining significant results from the Friedman test, we proceeded to conduct pairwise comparisons using the Durbin–Conover method. This method is suitable for post hoc analysis following a significant Friedman test and allows for the identification of specific group differences. Each pairwise comparison was adjusted for multiple comparisons to control the family-wise error rate. Effect sizes (*ESs*) were calculated to provide a measure of the magnitude of differences observed. For the Friedman test, Kendall’s W was used to evaluate the strength of the relationship between variables. It ranges from 0 to 1, with values close to 1 indicating a strong association, while values close to 0 indicate a weak or no association.

Next, a box plot was created to facilitate the interpretation of the statistical data. Physical and physiological metrics were standardized to ensure a uniform scale for comparison. The variables were represented across different training conditions (training matches, SSGs, and analytical tasks), differentiated by shades of gray, with the legend titled “Tasks” placed below the figure.

Following significant results from the Friedman test, pairwise comparisons were conducted using the Durbin–Conover method. Asterisks indicated significant differences between conditions. Additionally, gridlines were added, and median values were highlighted to enhance readability.

Finally, to further understand the impact of the variables, an impact analysis was conducted. This involved calculating the differences between conditions for each subject and examining the relationship of these differences with the “Offset”, a variable included in the dataset. Spearman’s rank correlation coefficients (ρ) were computed to assess the strength and direction of these relationships.

The statistical analyses were conducted using the R statistical software (v.4.4.0) for Windows, which was also used to create the figure. The level of significance for all tests was set at *p* < 0.05.

## 3. Results

### 3.1. Descrptive Results of Anthropometric Data and Training Intensities Variables

Table 2 presents anthropometric and physical–physiological intensity data for 55 subjects in four conditions: total, training matches, SSGs, and analytical tasks. The anthropometric data, which were normally distributed and expressed as mean and standard deviation, included chronological age (15.54 ± 0.53 years), maturation age (14.36 ± 0.44 years), offset (1.17 ± 0.61), weight (62.83 ± 8.06 kg), height (171.63 ± 6.12 cm), and sitting height (87.55 ± 3.70 cm). The physical–physiological intensity data, which were not normally distributed and expressed as median and interquartile range (*IQR*), showed that the median Dist (m/min) varied, being 77.35 (14.61) in total, 83.09 (14.71) in training matches, 68.80 (15.49) in SSGs, and 80.10 (20.61) in analytical tasks. The Speed Max (km/h) was 16.62 (5.17) km/h in total, 22.98 (4.81) km/h in training matches, 21.82 (4.35) km/h in SSGs, and 15.68 (2.54) km/h in analytical tasks. The iTL showed an HR Avg (/min) of 145.00 (29.00) in total, 157.00 (14.00) in training matches, 153.31 (20.04) in SSGs, and 145.00 (22.96) in analytical tasks.

### 3.2. Comparison of Intensity Variables According to Task Type

Firstly, in Table 3, the Friedman test did not show significant effects for Acc (+2/min) (*Q* = 5.05, *p* = 0.079, *ES* = 0.05) or for Dec (+2/min) (*Q* = 6.87, *p* = 0.034, *ES* = 0.06). Consequently, no further pairwise comparisons were necessary for these variables.

For Dist (m/min), the Friedman test indicated a significant effect (*Q* = 26.22, *p* < 0.0001), with an *ES* of 0.24. Pairwise comparisons revealed that the Dist (m/min) was significantly greater during training matches compared to both SSGs (*Z* = 5.74, *p* < 0.0001) and analytical tasks (*Z* = 3.68, *p* < 0.0001). Additionally, Dist (m/min) was significantly less during SSGs compared to analytical tasks (*Z* = 2.06, *p* < 0.05).

Similarly, % HIA Rel showed a significant overall effect (*Q* = 43.07, *p* < 0.0001), with an *ES* of 0.39. Pairwise comparisons indicated that % HIA Rel was significantly greater during training matches compared to analytical tasks (*Z* = 7.63, *p* < 0.0001) and during SSGs compared to analytical tasks (*Z* = 6.72, *p* < 0.0001). However, no significant difference was found between training matches and SSGs (*Z* = 0.905, *p* = 0.341). Furthermore, regarding % HIA Abs, the Friedman test showed a significant effect (*Q* = 41.89, *p* < 0.0001), with an *ES* of 0.38. Pairwise comparisons revealed significant differences between training matches and analytical tasks (*Z* = 6.84, *p* < 0.0001) and between SSGs and analytical tasks (*Z* = 7.26, *p* < 0.0001). No significant difference was found between training matches and SSGs (*Z* = 0.41, *p* = 0.337).

Sprints Rel also showed a significant overall effect (*Q* = 28.35, *p* < 0.0001), with an *ES* of 0.26. Significant pairwise comparisons indicated that Sprints Rel was greater during training matches compared to analytical tasks (*Z* = 4.40, *p* < 0.0001) and during SSGs compared to analytical tasks (*Z* = 5.89, *p* < 0.0001). There was no significant difference between training matches and SSGs (*Z* = 1.49, *p* = 0.136). Moreover, for Sprints Abs, the Friedman test showed a significant effect (*Q* = 28.77, *p* < 0.0001), with an *ES* of 0.27. Pairwise comparisons revealed that Sprints Abs was significantly greater during training matches compared to analytical tasks (*Z* = 5.15, *p* < 0.0001) and during SSGs compared to analytical tasks (*Z* = 5.54, *p* < 0.0001). There was no significant difference between training matches and SSGs (*Z* = 0.40, *p* = 0.341).

Additionally, Speed Max (km/h) showed a significant effect (*Q* = 60.65, *p* < 0.0001), with an *ES* of 0.55. Pairwise comparisons indicated that Speed Max (km/h) was significantly greater during training matches compared to SSGs (*Z* = 2.54, *p* < 0.05) and analytical tasks (*Z* = 11.00, *p* < 0.0001). Speed Max (km/h) was also significantly greater during SSGs compared to analytical tasks (*Z* = 8.46, *p* < 0.0001). For Speed Avg (km/h), the Friedman test also showed a significant effect (*Q* = 24.04, *p* < 0.0001), with an *ES* of 0.22. Pairwise comparisons revealed that Speed Avg (km/h) was significantly greater during training matches compared to SSGs (*Z* = 4.06, *p* < 0.0001) and analytical tasks (*Z* = 5.24, *p* < 0.0001). There was no significant difference between SSGs and analytical tasks (*Z* = 1.18, *p* = 0.237).

PL/min showed a significant overall effect (*Q* = 6.76, *p* < 0.05), with an *ES* of 0.06. Pairwise comparisons revealed that PL/min was significantly greater during training matches compared to SSGs (*Z* = 2.45, *p* < 0.05) and during analytical tasks compared to SSGs (*Z* = 2.11, *p* < 0.05). There was no significant difference between training matches and analytical tasks (*Z* = 0.34, *p* = 0.933).

HR Max (/min) showed a significant overall effect (*Q* = 51.92, *p* < 0.0001), with an *ES* of 0.47. Pairwise comparisons indicated that HR Max (/min) was significantly greater during training matches compared to analytical tasks (*Z* = 9.23, *p* < 0.0001). HR Max (/min) was also significantly greater during SSGs compared to analytical tasks (*Z* = 7.54, *p* < 0.0001). Furthermore, HR Avg (/min) also showed a significant overall effect (*Q* = 25.30, *p* < 0.0001), with an *ES* of 0.23. Pairwise comparisons revealed that HR Avg (/min) was significantly greater during training matches compared to SSGs (*Z* = 2.60, *p* < 0.05) and analytical tasks (*Z* = 5.68, *p* < 0.0001). HR Avg (/min) was also significantly greater during SSGs compared to analytical tasks (*Z* = 3.08, *p* < 0.001).

**Figure 2:**
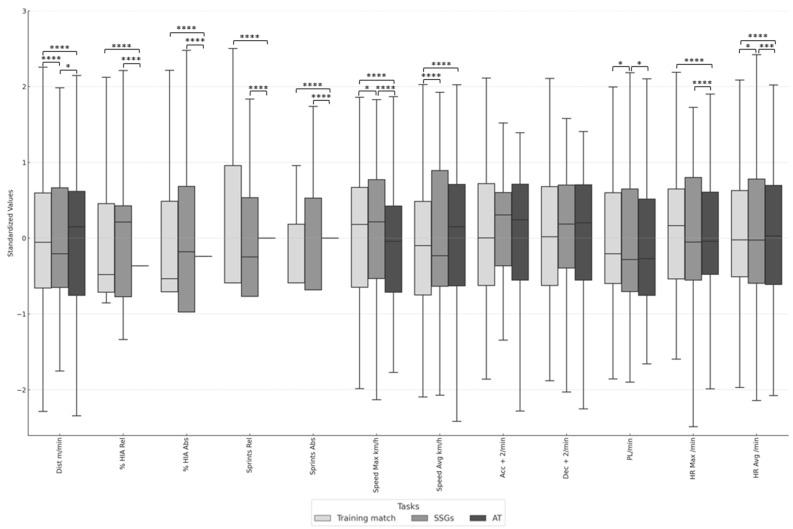
Comparison of standardized values of different variables across various training tasks. Note: SSGs = small-sided games; AT = analytical task. **** *p* < 0.0001, *** *p* < 0.001, * *p* < 0.05.

Figure 2 illustrates the comparison of how the standardized values of different variables vary across three training conditions (training matches, SSGs, and analytical tasks). The figure helps identify differences in the intensity and type of effort exerted by the players in each training task. Different shades of gray differentiate the conditions. Higher boxes in the plot indicate higher standardized values, suggesting greater relative intensity in that specific variable for the given context and vice versa. Likewise, the asterisks in the figure indicate significant differences between training tasks. The more asterisks there are, the greater our confidence that the observed difference between the training contexts is real and not due to random variations in the data, like those presented in Table 3. Comparisons without asterisks indicate that the differences are not statistically significant. This visualization underscores the need to tailor training sessions to address the varying intensities experienced during different training tasks. By understanding these differences, coaches and sports scientists can better design training sessions to optimize performance while minimizing the risk of injury.

### 3.3. Impact of Maturation Age on Differences between Study Contexts

The analysis of the Spearman’s correlation coefficient demonstrated that maturational age influenced certain variables related to eTL. Notably, significant correlations were observed in several aspects of players’ performance (Table 3).

Regarding % HIA Rel, the Spearman’s correlation coefficient revealed significant correlations between training matches and SSGs (ρ = 0.32, *p* < 0.05), as well as between training matches and analytical tasks (ρ = 0.33, *p* < 0.05). Similarly, for absolute % HIA Abs, significant correlations were found between training matches and SSGs (ρ = 0.32, *p* < 0.05) and between training matches and analytical tasks (ρ = 0.32, *p* < 0.05).

Acc (+2/min) showed significant correlations between training matches and SSGs (ρ = 0.30, *p* < 0.05). Dec (+2/min) also exhibited significant correlations between training matches and SSGs (ρ = 0.318, *p* < 0.05). Furthermore, PL/min demonstrated a significant negative correlation between training matches and analytical tasks (ρ = −0.276, *p* < 0.05).

However, many other variables did not show significant correlations. For instance, there were no significant correlations in Dist (m/min), Sprints Rel, Sprints Abs, Speed Max (km/h), Speed Avg (km/h), HR Max (/min), and HR Avg (/min).

## 4. Discussion

Training young athletes, especially in demanding sports like soccer, requires a careful and adaptive approach that considers variations in biological maturity. During the PHV period, it is crucial to adjust training intensity to optimize performance and minimize injury risk. This study monitored and compared eTL and iTL according to the type of training task (i.e., analytical tasks, SSGs, and training matches). Similarly, the effect of these training tasks on the maturational age of young soccer players was analyzed. The main results reported no significant differences for acceleration or deceleration per minute between training tasks. Pairwise comparisons reported significant differences in favor of training matches in Dist (m/min), Speed Max, Speed Avg, and HR Avg compared to SSGs and analytical tasks. In addition, % HIA and Sprints (in relative and absolute value) were significantly higher during training matches compared to analytical tasks and during SSGs compared to analytical tasks. PL/min was significantly higher during training matches compared to SSGs and during analytical tasks compared to SSGs. Speed Max, HR Max, and HR Avg were also significantly higher during SSGs compared to analytical tasks.

Regarding the main results related to the variables and training tasks studied, maturational age has been shown to have a significant relationship with certain metrics studied. In this sense, positive correlations with maturational age were observed for % HIA, Acc (+2/min), and Dec (+2/min) in training matches and SSGs, indicating more mature players engage in higher intensity activities. PL/min showed significant differences between conditions, with a negative correlation with maturational age, suggesting more mature players manage training intensities more efficiently. Additionally, no significant differences were found in Dist (m/min), Sprints, Speed Max, Speed Avg, HR Max, and HR Avg, indicating that other factors beyond biological maturation influence these performance variables.

### 4.1. Intensity Variables According to Task Type

Given the potential significance of acceleration movements as indicators of fatigue in soccer, the scientific literature suggests that SSGs could impose demands regarding acceleration capabilities higher than those experienced during full match play [42]. However, in this study, there were no significant differences in Acc (+2/min) or Dec (+2/min) according to the type of training task.

Our results reported significant differences in favor of training matches (11 vs. 11 full games) in Dist (m/min), Speed Max, and Speed Avg compared to SSGs and analytical tasks. Speed Max was also significantly higher during SSGs compared to analytical tasks. These results were consistent, as greater distances and larger game spaces provide more opportunities to reach higher speeds [4]. In a previous study [43], the young players’ running activity (from U-8 to U-14) was also significantly higher on matches played in 11 vs. 11 game format compared to 7 vs. 7 and 9 vs. 9 game formats. Furthermore, in Hodgson et al.’s study [42], male soccer players covered more total distance in 4 vs. 4 SSGs played on medium- and large-sized fields than on small-sized fields. The smaller field size reduced the area of active play and required players to make faster decisions and execute technical skills more frequently; however, players’ potential to frequently accelerate and/or reach high running speeds was compromised [42]. The differences in total distance covered between full matches and SSGs were also reported in other studies [44,45]. Although, these differences were minimal in the values reported by Clemente et al. [45] with professional players. Integrating full 11 vs. 11 matches and varied SSGs into training optimizes physical preparation, enhances decision-making and technical skills, and helps prevent injuries by tailoring intensities to different field sizes and player levels.

PL/min was significantly higher during training matches compared to SSGs and during analytical tasks compared to SSGs. Our results differ from a previous study on professional soccer [45], which reported higher playing load in SSGs compared to full matches. In addition, in this study, % HIA and Sprints (in relative and absolute value) were significantly higher during training matches compared to analytical tasks and during SSGs compared to analytical tasks. However, there were no significant differences between the training matches and the SSGs, unlike another studies [44,45] with professional soccer players. Both studies reported higher sprint performance in the full matches compared to medium and large SSGs. Considering the soccer modality, Rábano-Munoz et al. [46] reported that during Soccer-11, young players showed significantly higher high- and very-high- intensity actions compared to Soccer-7. In line with our results, Brito et al. [43], in U-8 to U-14 soccer players, reported that as high-intensity running activity increased, the effect of game format decreased. It is recommended to incorporate full training matches to enhance player load and high-intensity actions, while using SSGs for targeted skill development and managing physical intensity effectively.

To conclude with the intensity variables, there were significant differences in favor of training matches in HR Avg compared to SSGs and analytical tasks. HR Max was higher in training matches compared to analytical tasks. HR Max and HR Avg were significantly higher during SSGs compared to analytical tasks. Along these lines, Gómez-Carmona et al. [22] indicated that U-18 players who played SSGs and official matches reported low-intensity movements (<14 km/h), and medium–high HR (80–90% HR MAX) in the SSGs. This is consistent with the results reported in Asci’s study [47], that as field size per player increased, intensity and involvement in SSGs decreased. On the other hand, Little and Williams [48] found in professional soccer players that, as field size and number of players in SSGs increased, their HR and % HR Max decreased. In the present study, the opposite occurred: the greater the cognitive involvement and game spaces, the greater the objective iTL. It is recommended to integrate full training matches to maximize HR intensity and cognitive involvement, while using SSGs to balance physical and cardiovascular demands effectively.

### 4.2. Maturation Age on Differences between Study Contexts

In the present study, biological maturation was observed to have a significant influence on various kinematic and neuromuscular intensity variables. This suggests that individual differences in the maturation process can explain both the differences and similarities observed under different experimental conditions (training tasks).

For variables related to high-intensity activity (HIA), no significant differences were found between training matches and SSGs. However, differences were noted between training matches and analytical tasks and between SSGs and analytical tasks. Additionally, significant positive correlations with maturational age were found in comparisons between training matches and SSGs and between training matches and analytical tasks. This suggests that maturation influences high-intensity activity under these specific conditions. These findings are consistent with the literature, which shows that more mature players engage in more high-intensity actions during matches [49] and cover greater distances at high speed, regardless of their position on the field [50]. A possible explanation for these results is that players with higher maturational age have better capacity for high-intensity activities due to their greater physical and physiological development. This could explain why there are no significant differences between training matches and SSGs in terms of high-intensity activity, as both types of training can be equally demanding for more mature players. However, the significant differences observed between training matches and analytical tasks, as well as between SSGs and analytical tasks, could be due to analytical tasks not providing the same level of physical and competitive stimulus as training matches or SSGs. More mature players tend to perform more high-intensity activities regardless of the type of training, highlighting the influence of maturation on physical performance.

For Acc (+2/min) and Dec (+2/min), a significant positive correlation with maturational age was found between training matches and SSGs for both variables. This suggests that maturation influences individual differences in both accelerations and decelerations under these specific conditions. These results align with the literature, which finds that the frequency of high-intensity actions is greater in players participating in bio-banded tournaments [51]. Additionally, maturation can lead to greater gains in change in direction ability, especially in older subjects [52], as more mature players show better performance in tasks requiring high intensity [53]. For example, more mature players may have greater explosive capacity and control of rapid movements due to their advanced physical development. This could explain why, despite no significant differences in accelerations and decelerations between the different conditions, there is a positive correlation with maturational age. More mature players might better exploit game situations to accelerate and decelerate more effectively, excelling in scenarios that require rapid changes in pace, such as SSGs and training matches.

PL/min showed significant differences between conditions, with significant differences between training matches and SSGs and between SSGs and analytical tasks. Additionally, a significant negative correlation with maturational age was found between training matches and analytical tasks, suggesting that intensity decreases (they move less) with increased maturation in this specific comparison. This interpretation is supported by several studies analyzing how biological maturation affects intensity in young athletes. Teixeira et al. [54] demonstrated that chronological age, relative age, and biological maturation status significantly impact accumulated training intensity and perceived exertion. Similarly, other studies have found that maturation status influences session intensity perception and neuromuscular performance [55], while others have observed how it affects and accumulates training intensities throughout the season [56]. Together, these studies highlight that more mature players manage and adapt to training demands more efficiently, suggesting a critical role of biological maturation in physical performance and intensity management. From a practical standpoint, more mature players may better manage their effort during training matches and SSGs, allowing them to maintain a more consistent intensity adapted to their physical capacity. The negative correlation observed in analytical tasks could indicate that more mature players are more efficient in their performance during these exercises, needing less relative effort to achieve the same results as less mature players. This highlights how maturation can influence players’ ability to handle different types of intensity, better adapting to the requirements of each type of training task.

On the other hand, no significant differences were found in Dist (m/min), Sprints, Speed Max, Speed Avg, HR Max, and HR Avg. This suggests that performance in situational sports during adolescence may be influenced by factors additional to biological maturation. Factors such as physical activity levels, situational aspects of the task, general physical condition, technique and skill, and nutrition and recovery can play an important role. Furthermore, elements like previous training intensity, training environment, puberty adaptations, sports diversification, and genetic differences can also contribute to the lack of significant differences in these variables concerning maturation.

Factors like physical activity levels, situational aspects of the task, general physical fitness, technique and skill, and nutrition and recovery can play an important role [57,58,59]. Variations in physical activity levels among adolescents can mask differences due to biological maturation, especially in general physical performance measures [32]. Moreover, differences in physical performance may depend more on contextual and situational factors, such as team strategy, playing style, and field position, rather than biological maturation [60]. Motor learning and experience, through feedforward processes involving the anticipation and planning of movements based on previous experiences, can compensate for differences in biological maturation. Adolescents with more experience and better anticipation skills may show similar performance in these physical variables, regardless of their maturational age [61]. There is considerable inter-individual variability in response to physical stimuli and performance capacity, which can mask the effects of biological maturation [62]. Specific training adaptations can equalize differences in biological maturation, playing a crucial role in equalizing performance among players with different levels of maturation [63].

For these reasons, it is necessary to design specific and individualized training tasks according to the maturational age of the players. For example, when designing high-intensity activities that require constant changes in direction, more mature players may respond more efficiently compared to their teammates, and their performance may also be higher. This statement coincides with what was reported by Sampaio et al. [64], who also point out that football training programs should be based on the individual characteristics of the players, who can be grouped according to their similarities.

### 4.3. Limitations, Strengths, and Research Prospective

This study presents several limitations that should be considered. First, the sample size is limited, focusing on only two soccer clubs. Including more participants from various categories and levels would allow for more robust inter-group comparisons and generalizable results. Additionally, the generalizability of the findings may be restricted, as the sample may not represent all youth soccer contexts. Expanding the sample to different regions and levels of competition would provide a more comprehensive understanding. The controlled nature of training conditions introduces variability due to uncontrolled factors like weather and players’ physical state. Future studies could benefit from more standardized training environments. The measurement of maturational age based on anthropometric methods may lack accuracy. Using direct methods, such as radiographs, could improve measurement precision. The measurement technology used, despite being advanced, can suffer from technical issues. Ensuring proper calibration and maintenance of devices like IMUs and HR monitors is essential for data accuracy. Finally, it would be advisable to increase the number of sessions per team to at least four weeks [4]. Although the tasks analyzed in this training session can serve as a reference point, increasing the number of training sessions would provide a better balance between data accuracy and analytical feasibility.

This study’s strengths include a comprehensive analysis of physical and physiological intensities that reflect the demands of full matches, SSGs, and analytical tasks, highlighting the significant role of biological maturation in performance. It integrates multiple factors such as physical activity, technique, and situational contexts, providing a holistic view of adolescent sports performance. Emphasizing individual differences and acknowledging non-significant results further demonstrates a nuanced understanding, offering valuable insights for planning effective physical development and training in young athletes.

Future research should address these limitations and recommendations by expanding the sample size to improve generalizability, diversifying training tasks to analyze different exercise impacts, and implementing longitudinal studies to observe the long-term effects of biological maturation on performance. Using advanced measurement methods for maturational age and investigating the role of psychological and social factors will provide deeper insights into how these variables interact with training intensities and influence young players’ development. In summary, these future research directions will enhance the understanding of biological maturation’s influence and help optimize training practices to improve youth athletes’ performance.

## 5. Conclusions

The physical and physiological intensities of the participating players are affected by cognitive involvement and game spaces, which increase in full training matches. The results also highlight the significant influence of biological maturation on various physical variables, helping to explain the differences or similarities observed in the studied tasks. More mature players tend to engage in high-intensity actions more frequently and efficiently and manage training intensities more effectively. However, the lack of significant differences in variables such as distance covered per minute, maximum and average speed, and maximum and average heart rate suggests that other factors, such as physical activity levels, technique, recovery, and situational and contextual aspects, also could play a crucial role in sports performance during adolescence. Specifically, these findings emphasize the importance of considering biological maturation when evaluating and planning physical development and performance in young athletes.

## Figures and Tables

**Table 1:** Training intensities used in this study.

Intensity Type	Variable	Abbreviation	Description
Kinematic eTL	Distance	Dist m/min	Distance covered in meters per minute
	High-intensity actions in relative and absolute values	% HIA Rel % HIA Abs	It is the sum of the following variables: take-off (>3G), landings (>5G), impacts (>8G), accelerations (>3 m/s^2^), decelerations (<−3 m/s^2^), relative sprints (>95% max speed), and relative HSR (>75.5% max speed)
	Sprints in relative and absolute values	Sprints Rel	It is a displacement >21 km/h
		Sprints Abs	
	Maximum speed	Speed Max km/h	Maximum and average speed determined in kilometers per hour (km/h)
	Average speed	Speed Avg km/h	
	Accelerations	Acc + 2/min	Average accelerations exceeding 2 m/s^2^, per minute
	Decelerations	Dec + 2/min	Average decelerations below −2 m/s^2^, per minute
Neuromuscular eTL	Player load	PL/min	Vector magnitude derived from triaxial accelerometry data
iTL	Maximum heart rate	HR Max/min	It is established using the arithmetic mean of beats per minute
	Average heart rate	HR Avg/min	It is established using the arithmetic maximum of beats per minute

Note: eTL = External Intensity; iTL = Internal Intensity.

**Table 2:** Anthropometric data and training intensities variables across different training tasks.

Category	Variable	Total	Training Match	SSGs	Analytical Task
**Anthropometric Data** *M* ± *SD*	Chronological Age	15.54 ± 0.53			
	Maturation Age	14.36 ± 0.44			
	Offset	1.17 ± 0.61			
	Weight	62.83 ± 8.06			
	Height	171.63 ± 6.12			
	Sitting Height	87.55 ± 3.70			
**Intensity type** *Mdn* (*IQR*)					
Kinematic eTL	Dist m/min	77.35 (14.61)	83.09 (14.71)	68.80 (15.49)	80.10 (20.61)
	% HIA Rel	2.50 (7.44)	3.17 (9.94)	5.50 (4.25)	0.00 (0.00)
	% HIA Abs	1.00 (6.38)	1.29 (8.94)	2.48 (5.17)	0.00 (0.00)
	Sprints Rel	0.00 (0.00)	0.00 (1.00)	0.20 (0.50)	0.00 (0.00)
	Sprints Abs	0.00 (0.00)	0.00 (1.00)	0.00 (1.00)	0.00 (0.00)
	Speed Max km/h	16.62 (5.17)	22.98 (4.81)	21.82 (4.35)	15.68 (2.54)
	Speed Avg km/h	5.89 (0.94)	5.97 (1.12)	5.33 (1.20)	5.59 (0.84)
	Acc + 2/min	31.00 (5.33)	31.44 (5.08)	33.45 (3.37)	31.58 (6.42)
	Dec + 2/min	31.00 (5.34)	31.50 (4.95)	33.14 (4.05)	31.32 (6.34)
Neuromuscular eTL	PL/min	1.17 (0.35)	1.17 (0.30)	1.08 (0.34)	1.17 (0.38)
iTL	HR Max/min	164.00 (26.00)	186.00 (13.50)	180.00 (17.50)	164.00 (18.38)
	HR Avg/min	145.00 (29.00)	157.00 (14.00)	153.31 (20.04)	145.00 (22.96)

Note: *M* = mean; *SD* = standard deviation; *Mdn* = median; *IQR* = interquartile range; eTL = External Intensity; iTL = Internal Intensity; HIAs = high-intensity actions; Acc = acceleration; Dec = deceleration; PL = player load; HR = heart rate.

**Table 3:** Inferential results for eTL and iTL variables.

Variable	*Q*	*ES*	Comparison	*Z*	ρ
Dist m/min	26.22 ****	0.24	1 vs. 2	5.74 ****	−0.06 ns
			1 vs. 3	3.68 ****	−0.26 ns
			2 vs. 3	2.06 *	−0.19 ns
% HIA Rel	43.07 ****	0.39	1 vs. 2	0.91 ns	0.32 *
			1 vs. 3	7.63 ****	0.33 *
			2 vs. 3	6.72 ****	0.01 ns
% HIA Abs	41.89 ****	0.38	1 vs. 2	0.41 ns	0.32 *
			1 vs. 3	6.84 ****	0.32 *
			2 vs. 3	7.26 ****	−0.02 ns
Sprints Rel	28.35 ****	0.26	1 vs. 2	1.49 ns	0.09 ns
			1 vs. 3	4.40 ****	0.10 ns
			2 vs. 3	5.89 ****	0.06 ns
Sprints Abs	28.77 ****	0.27	1 vs. 2	0.40 ns	0.16 ns
			1 vs. 3	5.15 ****	0.06 ns
			2 vs. 3	5.54 ****	−0.08 ns
Speed Max (km/h)	60.65 ****	0.55	1 vs. 2	2.54 *	0.25 ns
			1 vs. 3	11.00 ****	0.15 ns
			2 vs. 3	8.46 ****	−0.07 ns
Speed Avg (km/h)	24.04 ****	0.22	1 vs. 2	4.06 ****	0.15 ns
			1 vs. 3	5.24 ****	0.11 ns
			2 vs. 3	1.18 ns	−0.03 ns
Acc +2/min	5.05 ns	0.05	1 vs. 2	1.55 ns	0.30 *
			1 vs. 3	0.68 ns	0.12 ns
			2 vs. 3	2.23 ns	−0.06 ns
Dec +2/min	6.87 *	0.06	1 vs. 2	1.76 ns	0.32 *
			1 vs. 3	0.88 ns	0.11 ns
			2 vs. 3	2.63 ns	−0.04 ns
PL/min	6.76 *	0.06	1 vs. 2	2.45 *	0.05 ns
			1 vs. 3	0.34 ns	−0.28 *
			2 vs. 3	2.11 *	−0.24 ns
HR Max/min	51.92 ****	0.47	1 vs. 2	1.69 ns	0.12 ns
			1 vs. 3	9.23 ****	−0.15 ns
			2 vs. 3	7.54 ****	−0.19 ns
HR Avg/min	25.30 ****	0.23	1 vs. 2	2.60 *	0.08 ns
			1 vs. 3	5.68 ****	−0.17 ns
			2 vs. 3	3.08 ***	−0.25 ns

Note: *Q* = Friedman test; *Z* = Durbin–Conover pairwise comparison statistic; ρ = Spearman correlation coefficient. Acc +2/min = accelerations per minute; Dec +2/min = decelerations per minute; PL/min = player load per minute; HR Max/min = maximum heart rate (beats per minute); HR Avg/min = average heart rate (beats per minute). 1 = training match, 2 = SSGs, 3 = analytical task. **** *p* < 0.0001, *** *p* < 0.001, * *p* < 0.05. ns = not significant.

## Data Availability

Data will be available upon reasonable request to the corresponding author.
